# Time‐restricted feeding improves the reproductive function of female mice via liver fibroblast growth factor 21

**DOI:** 10.1002/ctm2.195

**Published:** 2020-10-04

**Authors:** Lun Hua, Bin Feng, Liansu Huang, Jing Li, Ting Luo, Xuemei Jiang, Xingfa Han, Lianqiang Che, Shengyu Xu, Yan Lin, Zhengfeng Fang, De Wu, Yong Zhuo

**Affiliations:** ^1^ Institute of Animal Nutrition Sichuan Agricultural University Chengdu P. R. China; ^2^ Key Laboratory for Animal Disease‐Resistant Nutrition of the Ministry of Education of China Sichuan Agricultural University Chengdu P. R. China; ^3^ Key Laboratory of Animal Disease‐Resistant Nutrition of Sichuan Province Sichuan Agricultural University Chengdu P. R. China; ^4^ School of Life Sciences Sichuan Agricultural University Chengdu P. R. China; ^5^ Department of Pharmacology UT Southwestern Medical Center Dallas Texas USA

**Keywords:** fertility, FGF21, GnRH, time‐restricted feeding

## Abstract

**Background:**

There has been a significant increase, to epidemic levels, of obese and overweight women of reproductive age, causing impairments to reproductive health. Time‐restricted feeding (TRF) including isocaloric intake has shown to be preventive of obesity‐related disorders. However, its therapeutic ability to improve the reproductive function of female remains largely unknown.

**Methods:**

Here, we investigated the ability of TRF to improve the reproductive function in wild‐type and liver‐specific FGF21 knockout female mice. To study fertility, a continuous and a short‐term fertility test, gonadotropin releasing‐hormone (GnRH), and Kisspeptin test were performed. Immortalized GnRH neuron was used to examine the direct role of liver fibroblast growth factor 21 (FGF21) on GnRH secretion.

**Results:**

We found that TRF rescues female mice from bodyweight gain and glucose intolerance, as well as ovarian follicle loss and dysfunction of estrus cyclicity induced by high‐fat diet. Furthermore, the beneficial effects of the TRF regimen on the reproductive performance were also observed in mice fed both chow and high‐fat diet. However, those beneficial effects of TRF on metabolism and reproduction were absent in liver‐specific FGF21 knockout mice. In vitro, FGF21 directly acted on GnRH neurons to modulate GnRH secretion via extracellular regulated protein kinases (ERK_1/2_) pathway.

**Conclusions:**

Overall, time‐restricted feeding improves the reproductive function of female mice and liver FGF21 signaling plays a key role in GnRH neuron activity in female mice.

## Graphical abstract

1. Time‐restricted feeding increased fertility.
2. Time‐restricted feeding prevented hypothalamic hypogonadism.
3. FGF21 modulated GnRH secretion via ERK1/2 pathway.
4. Liver FGF21 signaling was involved in the beneficial effect of time‐restricted feeding on fertility.

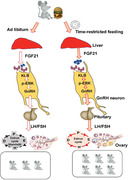

## 1 BACKGROUND

The prevalence of overweight and obesity among women of reproductive age is increasing.^1^, ^2^ Obesity has a detrimental effect on female health, and of which a particular concern was paid on the reproductive disorders.^3^ It has been demonstrated that in obese women, the “hypothalamic‐pituitary‐ovarian axis” was perturbed, and frequently present menstrual dysfunction, as well as anovulation and infertility.^2^, ^4^ The therapeutic intervention has largely involved prescribed lifestyle modifications including caloric restriction (CR) and increasing exercise, which is the common choice of anti‐obesity.^5^ Unfortunately, the sudden and significant restriction of caloric intake combined with increased exercise, while reducing body weight, may cause a stress response that alters hormone levels and damages ovarian follicle development, preserve ovarian follicle pool.^6^, ^7^ Recently, time‐restricted feeding (TRF), a daily eating pattern without altering nutrient quantities but limits the intake time‐period (usually less than 12 h) every day, has been demonstrated to reduce body weight, improve insulin sensitivity, and even longevity.^8^, ^9^, ^10^ However, the effect of TRF on reproductive functions remains poorly understood.

The reproductive hormones gonadotropin releasing‐hormone (GnRH), luteinizing hormone (LH), follicle‐stimulating hormone (FSH), and estrogen play a central role in maintaining the normal function of many reproductive processes. The major player in the system is GnRH, which is synthesized and released from the anterior and medio‐basal hypothalamus.^11^ GnRH is released in a pulsatile fashion and exerts its action on gonadotropic cells in the anterior pituitary leading to the release of gonadotropins (LH and FSH), which in turn stimulate gametogenesis and sex steroid production in the gonads.^12^ Disruption or perturbation of GnRH synthesis and secretion leads to hypogonadotropic hypogonadisms, which lead to subfertility and even infertility.^13^, ^14^

Modern lifestyles encourage extending the period of daily energy intake and thereby shortening the fasting period. This eating pattern has been associated with an increased prevalence of metabolic diseases.^15^ Clinical studies performed in overweight individuals undergoing TRF have shown a reduction in body weight, insulin resistance, and serum cholesterol concentration through the increased expression of thermogenic genes.^10^, ^16^ FGF21 was first discovered as a fast‐response hormone, and was later observed to be induced by a variety of nutritional situations such as a low‐protein diet, a ketogenic diet, and alcohol consumption.^17^, ^18^, ^19^, ^20^, ^21^ FGF21 signals to several target tissues, including the brain,^22^, ^23^ adipose tissues,^24^, ^25^, ^26^ and pancreas^27^ through a receptor complex consisting of the FGF receptor (FGFR1c), and the co‐receptor β‐klotho.^28^ FGF21 can induce thermogenesis and energy expenditure by promoting white adipose tissue browning and the activation of brown adipose tissue.^24^, ^25^, ^26^, ^29^, ^30^ Both FGFR1c and β‐klotho are expressed in hypothalamic neurons^31^, ^32^ and FGF21 has shown a non‐saturable, unidirectional influx across the blood‐brain barrier.^31^, ^32^, ^33^ Injection of FGF21 to rats by intracerebroventricular increased energy expenditure and insulin sensitivity.^26^, ^32^ Meanwhile, FGF21 has been shown to have an effect on in maintaining glucose homeostasis through the central nervous system.^32^ Furthermore, β‐klotho knockout mice exhibited delayed puberty, cyclicity disruption, and subfertility with impaired GnRH functioning.^31^ These results suggested that the hepatokine FGF21, a key central metabolic regulator, may play a role in linking metabolism to reproductive outcomes.

Time‐restricted feeding is being considered as a potential therapy for humans with obesity and related metabolic syndromes.^34^ Considering the unique physiologic features of female reproduction that is more sensitive to the environment, we were cautious regarding the positive effects of Time‐restricted feeding observed in males. In the current study, we found that TRF rescues female mice from body weight gain and glucose intolerance, as well as ovarian follicle loss and dysfunction of estrus cyclicity induced by high‐fat diet. Furthermore, TRF induced liver FGF21 secretion, which acts as an endocrine signal and plays a key role in GnRH neuron homeostasis, therefore, improves the reproductive function in female mice. Taken together, our findings provide evidence that during TRF, FGF21 potentially acts as an endocrine signal linking metabolism to reproductive health in females.

## 2 MATERIALS AND METHODS

### 2.1 Animals

In this study, all the animal procedures were approved by the Institutional Animal Care and Research Committee of Sichuan Agricultural University. Eight‐week‐old C57BL/6J female and male mice from Vital River Laboratory Animal Technology Co. Ltd. (Beijing, China). The FGF21 liver‐specific knockout (FGF21LKO) mice have been previously characterized.^29^, ^35^ Briefly, FGF21^loxp/loxp^ mice from Jackson Laboratory (022361; Bar Harbor, ME, USA) were mated with Alb‐Cre mice from Nanjing University (J003574; Model Animal Research Center, Nanjing, China) to generate FGF21^loxp+/‐, Alb‐Cre^ mice. And then FGF21^loxp+/‐, Alb‐Cre^ mice were mated with FGF21^loxp/loxp^ mice to generate FGF21^loxp/loxp, Alb‐Cre^. FGF21^loxp/loxp^ mice littermates were used as controls. Mice were kept in 24 ± 2°C facilities with a 12‐h light: 12‐h dark schedule and had free access to water.

### 2.2 Feeding schedule and diets

Eight‐week‐old and body weight‐matched female C57BL/6J mice were fed with normal diet (D12450B; 10 kcal% from fat) or high‐fat diet (D12492; 60% kcal from fat) either with ad libitum or time‐restricted access to food. In the TRF regimen, only between 21:00 to 07:00 on the next day the mice were allowed to access food, by transferring mice daily between cages with water only and cages with food and water.^8^, ^9^ As a control for handling, *ad libitum* fed mice were also transferred between feeding cages at the same time. Bodyweight and food intake were measured weekly.

### 2.3 Glucose tolerance test

After overnight fasting, mice fed with normal diet or high‐fat diet were administered with d‐glucose at the dose of 1 g/kg body weight by intraperitoneal injection. Blood glucose levels were measured at 0, 15, 30, 45, 60, 90, and 120 min post‐injection with tail vein blood using glucose test strips.

### 2.4 Reproductive assessment

Estrus cyclicity was monitored for 15 consecutive days by daily cytological analysis of vaginal smears (n = 20). Identification of the estrus cycle stage was performed as previously described.^29^

After 8 week TRF treatment, a continuous 17‐week mating protocol was used to measure the number of litters per female and litter size as previously described.^36^ Confirmed fertile males and female mice were housed together (1:1) at 21:00 and were left with the females until 07:00 with randomly rotated. Each litter was sacrificed at birth to allow the dams to re‐enter estrus cyclicity within a few days.^36^ Reproductive assessment of a short‐term fertility study was conducted as follows: Briefly, WT and FGF21LKO female mice housed together (1:1) were exposed to a confirmed fertile male. Males were randomly rotated among the cages at 21:00 and were left with the females until 07:00. During fertility testing, feeding schedules were kept the same as before breeding. The litter size, total number of offspring and time to litter were recorded.

Females were subjected to GnRH and Kisspeptin tests on the day of the first diestrus, and blood were collected before and after 30 min IP injections of either 0.25 μg GnRH or 1 nmole Kisspeptin (Kp‐10) per mouse, respectively.^31^ Blood samples were collected by tail puncture and stored at ‐80°C until LH measurement by ELISA.

### 2.5 Sample collection

After 22 or 24 weeks of dietary treatment, mice were euthanized by carbon dioxide, and followed cervical dislocation at different stages of the estrus cycle. The blood samples were centrifuged for 15 min at 3000 × *g* to collect serum and stored at ‐20°C for future analysis. Both ovaries were excised. The right‐side ovary was frozen in liquid N_2_ and stored at −80°C pending analysis of gene expression, whereas the other was fixed in 4% paraformaldehyde for histological examination. The brains were placed ventral side up, frontal sections of the hypothalamus (1 mm‐thick) were collected using a brain matrix (15003, Ted Pella, Inc.). The AVPV was micro‐dissected, processed for RNA extraction.

### 2.6 Culture of GT1‐7 cells

GT1‐7 cells were cultured under 5% CO_2_ at 37°C, with 10% fetal bovine serum (10099141; Thermo Fisher Scientific) in DMEM medium (11965092; Thermo Fisher Scientific). GT1‐7 cells were treated with rFGF21 (8409; Bio‐Techne, Minneapolis, MN, USA) with or without the ERK_1/2_ inhibitor U0126 (U120; Sigma, St. Louis, MO, USA; 10 μM). Inhibitors pretreated 30 min before rFGF21 treatment, the culture medium was collected after 12 h to measure GnRH levels by ELISA.

### 2.7 Analysis of hormones

Serum 17β‐estradiol (KGE014; R&D Systems) and FGF21 (MF2100; R&D Systems), adiponectin (EZMADP‐60K, Millipore), Leptin (EZML‐82K; Millipore), LH (KA2332; Novus Biologicals; Littleton; CO; USA) levels, and culture medium GnRH (B163244, BIM, San Francisco, CA, USA) levels were measured with commercial ELISA kits.

### 2.8 RNA extraction and gene expression analysis

RNA extraction and RT‐PCR were performed as previously reported.^29^, ^35^ Briefly, RNA from ovarian was extracted by Trizol (15596018; Thermo Fisher Scientific) and purified using RNA mini‐columns (RR037A; Takara Bio). RT–PCR was conducted in 10 μl of reaction volume with SYBR Green (RR820A; Takara Bio). The sequences of the primers are listed in Supplemental Table S1.

### 2.9 Ovaries histology and follicle counts

Ovaries were fixed with 4% paraformaldehyde, and then dehydrated and embedded in paraffin. Embedded ovaries were sliced into 5 μm sections. For H&E staining, every fifth sections were stained and examined by microscopy (Nikon 80i). The count of ovarian follicles was performed as previously described.^35^ Primordial, primary, secondary and antral follicles were quantitated by oocytes with visible nuclei. The total follicle counts were multiplied by a correction factor of 5 to represent the estimated number of follicles at each developmental stage.

### 2.10 Statistical analysis

Data were analyzed with GraphPad Prism 6 software (GraphPad Software, La Jolla, CA, USA). Normality tests were performed to confirm that data are normally distributed before Unpaired *t*‐test, ANOVA analysis, and Tukey's test were performed, after the Levene's test was performed to check homogeneity of variance by GraphPad Prism 6 software. Unpaired *t*‐test was used to analyze the difference between two groups. For the time of pregnancy, data were analyzed by Kaplan‐Meier statistics. For the experiments involving different diets or FGF21LKO mice, two‐way ANOVA test was used and the Tukey's test was performed for multiple comparisons between each group. Data are shown as mean ± SEM. Statistical significance was determined at *P *< .05.

## 3 RESULTS

### 3.1 TRF rescued female mice from high‐fat diet induced cyclicity dysfunction

**FIGURE 1:**
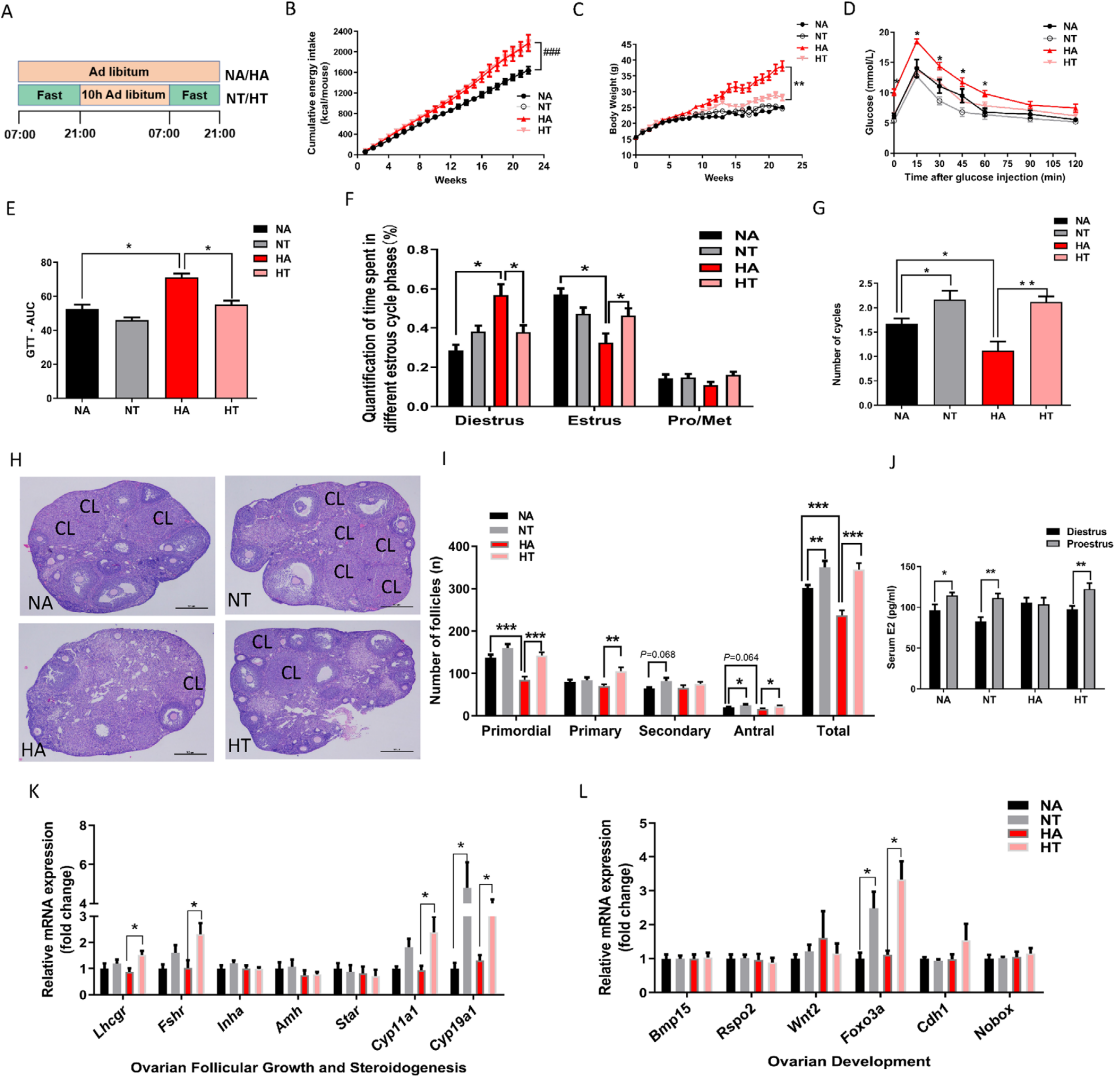
Time‐restricted feeding prevented body weight gain and menstrual dysfunction. (A) Schematic illustration of the experimental design. Female mice were fed with chow diet or high‐fat diet for 22 weeks with or without TRF treatment. NA means mice eating a normal chow diet with *ad libitum*, NT means mice eating a normal chow diet with time‐restricted access to food, HA means mice eating a high‐fat diet with *ad libitum*, and HT means mice eat a high‐fat diet with time‐restricted access to food. (B) Cumulative energy intake. (C) Body weight. (D) Glucose tolerance test and calculation of area under curve (E) were performed after overnight fast (n = 7). (F) Quantification of time spent in different estrus cycle phases and the number of cycles in the 15 days experimental length (G) (n = 20). Representative ovarian histology of mice after 22 weeks treatments (H) and follicle counts at each developmental stage in proestrus (I). (J) Serum estradiol (E2) concentrations at diestrus and proestrus. RT‐PCR analysis for mRNA expression levels related to ovarian follicular growth and steroidogenesis (K and L) at proestrus (n = 8). Data are means ± SEM. Statistical significance was evaluated by the 2‐way ANOVA test. Columns with different by the Tukey's test or unpaired t‐test. ^*^ *P* < .05, ^**^ *P* < .01, ^***^ *P* < 0.001

Previous studies reported that TRF prevented diet‐induced metabolic dysfunction in males.^8^, ^9^ To further evaluate whether TRF could prevent diet‐induced reproductive dysfunction in females, we investigated the consequences of TRF on metabolism in female mice. Eight‐week‐old female mice were fed either normal chow diet (ND) or a high‐fat diet (HFD) ad libitum or had time‐restricted access to food (10 h free access food daily) for 22 weeks (Figure 1A). Compared to mice fed on the ND‐ad libitum (NA), the ND‐TRF (NT) mice had similar cumulative energy intake (Figure 1B) and body weight (Figure 1C). Compared to mice fed on the HFD‐ad libitum (HA), HFD‐TRF (HT) mice had similar cumulative energy intake (Figure 1B), but had reduced body weight gain (Figure 1C). Circulating glucose clearance as indicated by a glucose tolerance test (GTT), a sign of insulin sensitivity, were improved in mice fed HFD with time‐restricted access to food as compared to the mice fed the HFD with ad libitum, but no difference in the NT fed mice compared to the NA fed mice (Figure 1D,E).

The obese female had a greater incidence of menstrual dysfunction and anovulation.^1^, ^2^, ^4^ To determine whether TRF could rescue female mice from the estrus cycle dysfunction caused by high‐fat diet, estrus cycles were continuously monitored by vaginal smear cytology. Interestingly, mice fed the high‐fat diet *ad libitum* exhibited irregular estrus cycles compared to their TRF counterparts, with a significant increase of the time spent in diestrus (Figure 1F,G). In addition, compared to mice fed the HA diet, HT‐fed mice exhibited a shorter cycle length (Figure 1G). These results indicated that TRF prevented the disorder of estrus cycle induced by HFD.

### 3.2 TRF promoted ovarian follicular development

Compared to the HA or NA fed mice, TRF increased the number of corpora lutea (Figure 1H). Ovarian follicle pool had a great impact on the reproductive span of female. The numbers of primordial follicle, antral follicle, and total follicle at all developmental stages were decreased by high‐fat feeding, but were increased by TRF (*P* < .05 or *P* < .01, Figure 1I). In NA, NT, and HT fed mice the circulating 17β‐estradiol (E2) levels underwent cyclic changes during the estrus cycle, which were absent in the HA fed mice (Figure 1J). The mRNA levels of ovarian gonadotropin receptors (*Lhcgr and Fshr*) and steroidogenic enzymes (*Cyp19a1*, cytochrome P450, family 19, subfamily A polypeptide 1 and *Cyp11a1*, cytochrome P450, family 11, subfamily A polypeptide 1), were elevated by the TRF regimen in mice fed the HFD, while no differences in the expression of inhibin (*Inhha*), steroidogenic acute regulatory protein (*Star*) and anti‐Mullerian hormone (*Amh*) mRNA were observed (Figure 1K). We next evaluated the genes related ovarian follicular development expression level,^36^ including bone morphogenetic protein 15 (*Bmp15*), Wnt family member 2 (*Wnt2*), R‐spondin 2 (*Rspo2*), cadherin 1 (*Cdh1*), forkhead box O3 (*Foxo3a*), and NOBOX oogenesis homeobox (*Nobox*). The mRNA expression of *Foxo3a* was elevated by TRF in mice fed the ND and HFD diets (Figure 1L). These analyses indicated that TRF improved ovarian development.

### 3.3 TRF increased fertility

**FIGURE 2:**
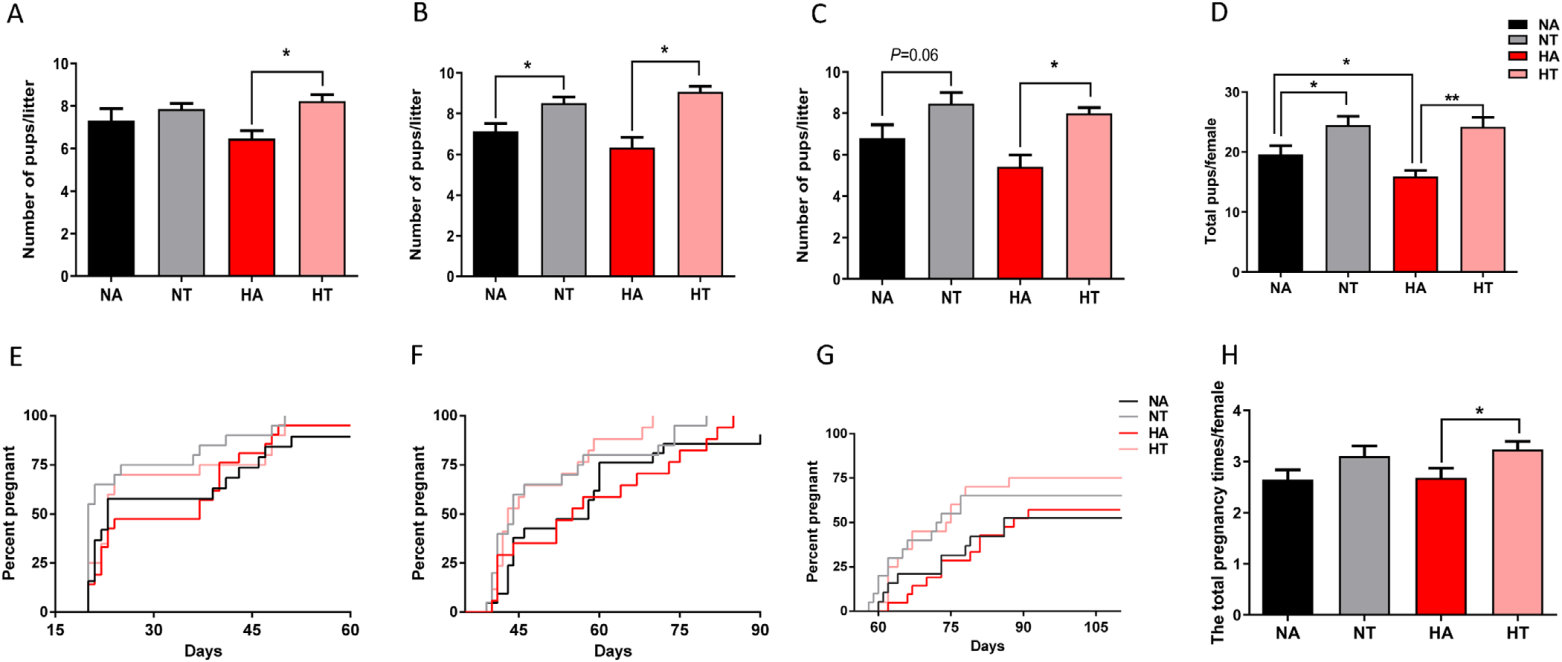
Time‐restricted feeding increased fertility. Female mice were fed with chow diet or high‐fat diet for a continuous mating protocol for 17 weeks after 8 weeks’ dietary treatment. The litters size of first pregnancy (A), second pregnancy (B), third pregnancy (C) and the total number of litters (D) per female. Data are means ± SEM; n = 20–21 per group. Statistical significance was evaluated by the two‐way ANOVA test. Columns with different letter * or ** denote *P* < 0.05 or *P* < 0.01 by the Tukey's test, respectively. The latency to pregnancy from male introduction in first pregnancy (E), second pregnancy (F) and third pregnancy (G) and the total pregnancy times of each female (H) during the 17 weeks’ mating period, the X axis is representing time to litter from male introduction. Data are means ± SEM; n = 20–21 per group; Statistical significance was evaluated by Kaplan Meier statistics

To determine the effects of TRF on the production of offspring, mice were subjected to a 17‐week continuous mating test after TRF treatment for 8 weeks. Compared to the HA fed mice, litter size was greater in the HT fed mice from the first to the third pregnancies (Figure 2A‐C). Moreover, the litter size of the NT fed mice in the second and the third pregnancies was also greater than the mice fed the NA diet (Figure 2B,C), while no difference in litter size was observed in the first pregnancies between mice fed the NA and NT diets (Figure 2A). The cumulative number of pups across three pregnancies was decreased in mice fed the high‐fat diet *ad libitum* compared to the chow‐fed mice (Figure 2D), but this decrease in the cumulative number of pups subjected to the high‐fat diet was rescued by the TRF regimen (Figure 2D). On average, mice fed the HA diet showed a delay for successful pregnancies compared to the HT fed mice from the second pregnancy to the third pregnancy (*P *< .05, Figure 2F,G), but no difference at the first pregnancy (*P *> .05, Figure 2E). HT‐fed mice had greater number of pregnancies and percentage of mice pregnant/round compared to the mice on the HA diet, while no difference was observed between the NA fed mice and the NT fed mice (Figure 2H; Table S2). These data suggested that TRF improved the female fertility in mice fed both normal and HFD.

### 3.4 TRF prevented hypothalamic hypogonadism induced by an HFD

**FIGURE 3:**
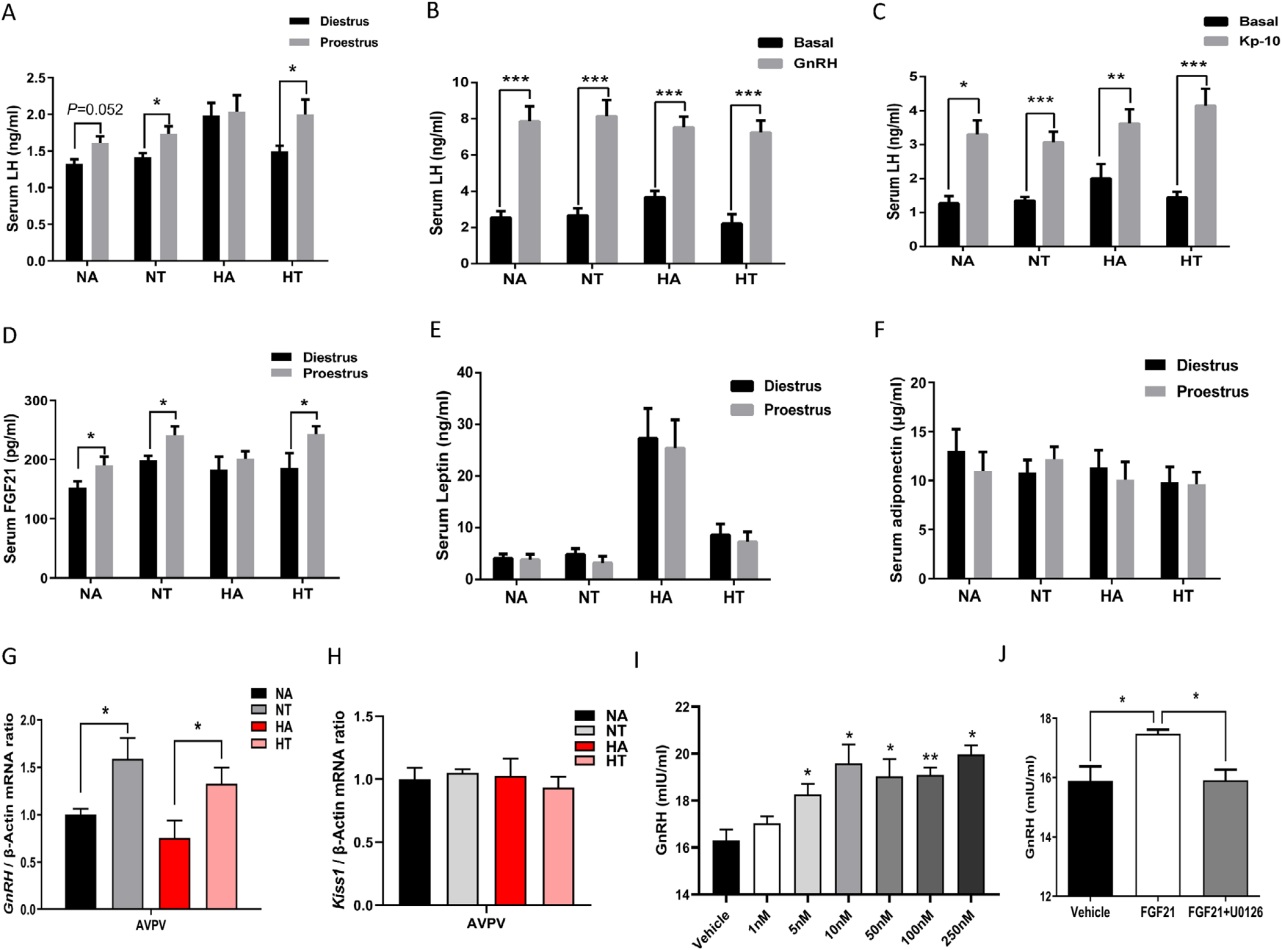
Time‐restricted feeding prevents hypothalamic hypogonadism. Female mice were fed with chow diet or high‐fat diet for 22 weeks with or without TRF treatment. (A) Serum LH concentrations at diestrus and proestrus; (B) GnRH test: blood LH levels at basal or 30 min after intraperitoneal GnRH injection (0.25 μg) in female mice; (C) Kisspeptin test: blood LH levels at basal and 30 min after intraperitoneal Kisspeptin (1 nmol) injection in female mice; Serum FGF21 (D), leptin (E), and adiponectin (F) levels at diestrus and proestrus; *Gnrh* (G) and *Kiss‐1* (H) mRNA expression levels in hypothalamus during proestrus, n = 6–9 /group. (I) Culture medium GnRH levels after rFGF21 treatment for 12h in GT1‐7 cell line; (J) Culture medium GnRH level, cells were pretreated with inhibitors (U0126) 30 min before rFGF21 (50 nM) treatment and were treated with rFGF21 plus inhibitors for 12h. Data are means ± SEM; Statistical significance was evaluated by Unpaired t‐test. *P < 0.05, **P < 0.01, ***P < 0.001

In order to investigate the effect of TRF on hypothalamic hormone secretion, blood samples were harvested at proestrus and diestrus. The serum LH concentrations at proestrus were greater in mice at proestrus than those at diestrus for mice fed the NA, NT, and HT diets, but the cyclic changes of LH between proestrus and diestrus were absent for mice fed the HA diet (Figure 3A), which suggested that TRF preserved the normal cyclic LH rise of mice fed the HFD. In females, the normal estrus cyclicity requires cyclic rise of hypothalamic GnRH secretion and a preovulatory LH surge to induce the oocyte development.^37^ To assess the function of the hypothalamic‐pituitary‐gonadal axis in female mice after 22 weeks of TRF treatments, we performed a series of hormone challenge tests as previously described.^23^, ^31^, ^38^ Intraperitoneal (IP) GnRH injections elicited a robust rise in LH in the four groups of mice (Figure 3B), which excluded a pituitary defect. Furthermore, IP Kisspeptin (Kp‐10) injections also induced LH secretion (Figure 3C) in all groups, suggesting that GnRH neurons are present and can respond to stimulation. We measured the peripheral hormones that may link metabolic status to reproduction. Serum FGF21 (Figure 3D), but not leptin and adiponectin (Figure 3E,F), underwent cyclic changes during the estrus cycle, similar to the changes in circulating LH levels (Figure 3C). These findings indicated that circulating FGF21 may link the regulation of TRF on central GnRH/LH secretion.

To test the hypothesis that TRF may increase hypothalamic GnRH secretion, we measured *Gnrh* mRNA levels in hypothalamic regions by RT‐PCR. Interestingly, we found that the mRNA expression levels of *Gnrh* in the anteroventral periventricular (AVPV) of hypothalamic was affected by TRF (Figure 3G). And in all groups, the hypothalamic *kiss‐1* mRNA levels were similar (Figure 3H). These results illustrated that the peripheral stimulation of GnRH secretion was enhanced by the TRF treatments, independent of Kisspeptin expression.

### 3.5 Liver FGF21 signaling was involved in the beneficial effect of time‐restricted feeding on fertility

**FIGURE 4:**
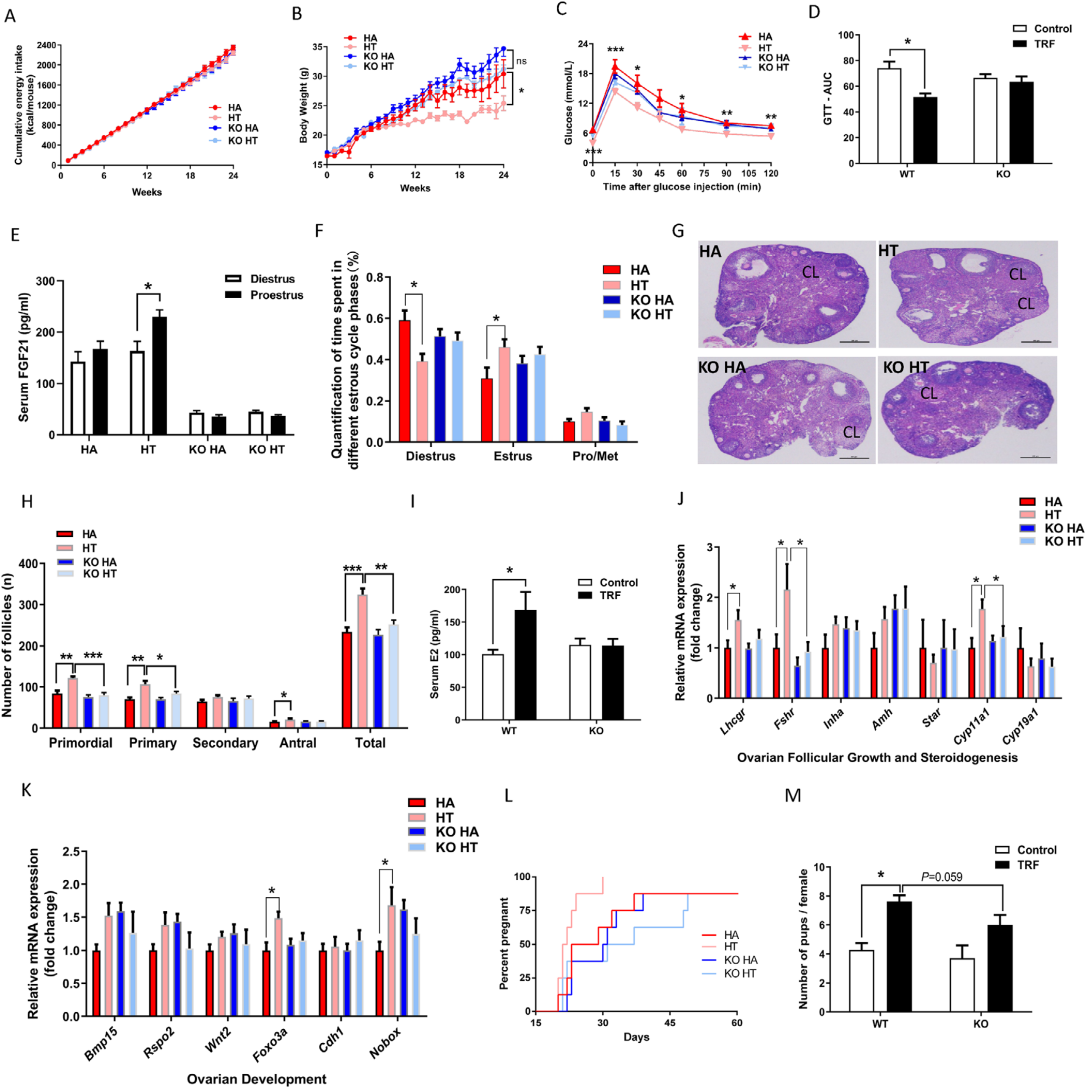
Liver FGF21 signaling was involved in the beneficial effect of time‐restricted feeding on fertility. WT or FGF21LKO mice were fed with high‐fat diet for 24 weeks with or without TRF treatments. (A) Cumulative energy intake. (B) Body weight. (C) Glucose tolerance test, and area under curve of glucose (D). (E) Serum FGF21 levels at diestrus and proestrus. (F) Quantification of time spent in different estrus cycle phases for a continuous 15‐day phase in 4 groups (n = 15). Representative picture of the ovaries (G) and follicle counts at each developmental stage per ovary in proestrus (H). (I) Serum 17β‐estradiol levels at proestrus. RT‐PCR analysis of mRNA expression levels of genes related to ovarian follicular growth and steroidogenesis (J) and ovarian development (K) at proestrus. A short‐term fertility study: the latency to pregnancy from male introduction in first pregnancy, the X axis is representing time to litter from male introduction (L) and the litter size (M). Data are means ± SEM; n = 6–9 / group unless stated; Statistical significance was evaluated by the 2‐way ANOVA test or with the Tukey's test for multiple comparisons to determine differences among each group, where appropriate. Statistical significance was evaluated by Kaplan Meier statistics for the latency to pregnancy. *P < 0.05, **P < 0.01, *** *P* < 0.001

Given that TRF increased serum FGF21 by 23% (*P* < .05; Figure 3D), and serum FGF21 underwent cyclic changes as similar to LH (Figure 3D). The direct effects of FGF21 on the secretion of GnRH were examined in immortalized GnRH secreting cell lines (GT1‐7 cells). FGF21 treatment resulted in a rise of GnRH levels in the culture medium after 12 h of treatment in a dose‐dependent manner (Figure 3I). To test whether the ERK_1/2_ pathway was required for FGF21 inducing GnRH secretion, GT1‐7 cells were treated with U0126 to block ERK_1/2_ signaling. Results showed that ERK_1/2_ inhibitor abolished FGF21 induced GnRH secretion (Figure 3J). These results demonstrated that FGF21 directly induced GnRH secretion through ERK_1/2_ signaling. Liver‐derived FGF21 is the main contributor to circulating FGF21,^29^, ^32^, ^35^ thus the FGF21LKO female mice model was used. To investigate the effect of fasting on *Fgf21* mRNA expression levels in liver, epididymal WAT (eWAT), inguinal white adipose tissue (iWAT), brown adipose tissue (BAT), and skeletal muscle, we measured *Fgf21* mRNA levels both in fed and fasted state. Indeed, liver *Fgf21* mRNA was induced by fasting in WT mice, but was completely abolished in FGF21LKO mice (Figure S1A). Whereas the *Fgf21* expression level showed no difference in eWAT (Figure S1B), iWAT (Figure S1C), BAT (Figure S1D), and skeletal muscle tissues (Figure S1E) either in fed or fasted state. Wild‐type and FGF21LKO mice were ad libitum or subjected to TRF for 24 weeks in HFD. The lack of circulating FGF21 levels was confirmed (Figure 4E), 79% of FGF21 in circulations were lost in FGF21LKO mice. Neither the genotype nor the feeding pattern affected food intake (Figure 4A). HFD‐TRF (HT) mice have similar cumulative food intake compared to HA mice (Figure 4A), but had reduced body weight (Figure 4B). Conversely, the lowering effects of TRF on body weight gain was lost in the FGF21LKO mice (Figure 4B). TRF treatment ameliorated HFD‐induced glucose in tolerance in the WT mice, which were not observed in the FGF21LKO female mice (Figures 4C,D). The serum FGF21 concentration at proestrus was decreased in mice fed the high‐fat diet, but not in HT mice (Figure 4E). The HA mice spent greater time at diestrus and less time at estrus than the HT mice, but no such differences were observed for the FGF21LKO mice (Figure 4F). The number of ovarian follicular development was affected (Figure 4G,H). The numbers of ovarian primordial follicle, primary follicle, antral follicle, and the total number of follicles at all developmental stages, were greater in HT mice that the HA mice, but no difference of such changes were observed between KOHA and KOHT mice (Figure 4H). Circulating concentrations of E2 was greater in mice fed the HT diet compared with mice fed the HA diet, but remained unchanged between the KOHA and KOHT mice (Figure 4I). Furthermore, the mRNA levels of ovarian gonadotropin receptors (*Lhcgr* and *Fshr*), the steroidogenesis enzyme gene (*Cyp11a1*), and the ovary development‐related genes (*Foxo3a*, *Nobox*) were upregulated by TRF in the WT mice, but not in the FGF21LKO mice (Figure 4J,K). In addition, the short‐term fertility test showed a delay to pregnancy (Figure 4L) and a decreased litter size (Figure 4M) in the HA fed mice compared with the HT fed mice, but not in the FGF21LKO mice, and the pups had a tendency to be decreased in FGF21LKO mice compared with WT mice under TRF regimen (*P *= .059, Figure 4M). These data suggest that liver FGF21 mediates the beneficial effects of the TRF on the metabolism and fertility.

## 4 DISCUSSION

**FIGURE 5:**
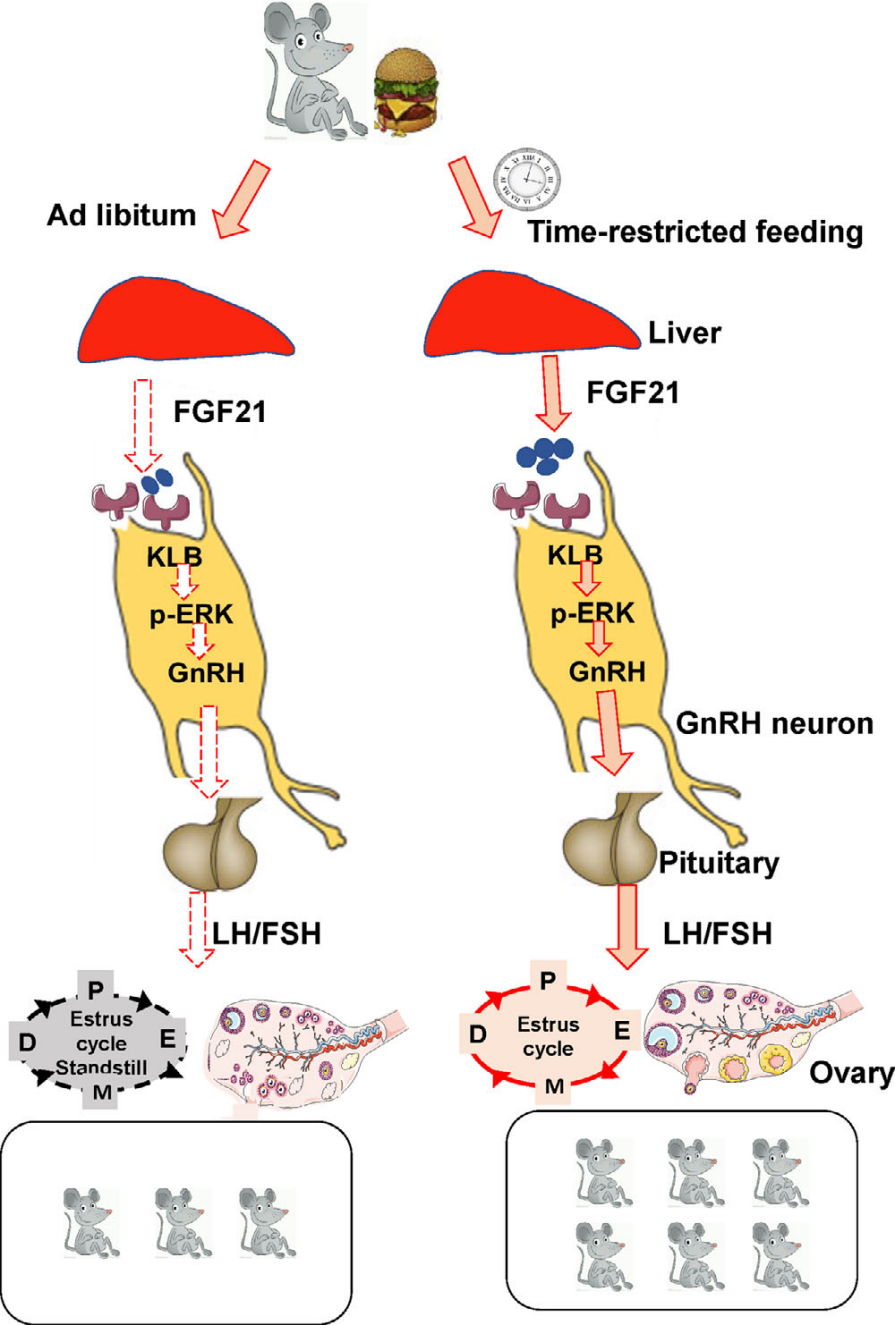
A working model of time‐restricted improves the reproductive function of female mice via liver FGF21. Time‐restricted feeding increased fertility and prevented hypothalamic hypogonadism. FGF21 directly acted on GnRH neurons to modulate GnRH secretion via ERK1/2 pathway. Liver FGF21 signaling was involved in the beneficial effect of time restricted feeding on fertility

Reproductive fitness is tightly linked to energy homeostasis, and both obesity or rapid weight loss can lead to reproductive disorders.^2^ Time‐restricted feeding, a feeding pattern that involves extended fasting, has been shown to protect against obesity and metabolic dysfunction.^34^ Here, we provided evidence that TRF can improve the reproductive function of female mice via FGF21 modulated GnRH secretion (Figure 5). Overall, the current findings of this study offered an interorgan physiological mechanism involving FGF21 signaling that potentially links metabolic and reproductive health.

Obesity is a common health problem that causes multiple adverse effects on reproductive function among women of reproductive age, including irregular estrus cycle, anovulation, polycystic ovarian syndrome, and even infertility.^39^ Time‐restricted feeding has been shown to protect against obesity related‐diseases, such as type II diabetes and nonalcoholic fatty liver disease.^8^, ^34^ Consistent with previous studies, we found TRF rescued females from the HFD‐induced body weight gain (Figure 1C) and glucose intolerance (Figure 1D,E) independent on caloric intake. In humans and mouse, feeding an HFD ad libitum dampens circadian rhythms, thereby causing metabolic damage.^40^ Disruption of circadian rhythms has been reported to be associative with poor fertility or even infertility.^41^ Interestingly, rhythmic change in expressions of circadian clock components is sustained when diet‐induced obesity mouse is subjected to TRF.^8^ In addition, TRF can prevent and even reverse obesity‐related metabolic disorders.^8^ One of the negative effects of obesity on female reproduction is hypogonadism caused by inhibited GnRH neuronal activity.^42^, ^43^ A high‐fat diet or obesity promotes ovarian macrophage infiltration and impairs female ovarian development and oocyte mature.^44^ A cyclic rise of reproductive hormones such as GnRH, FSH, and LH is required for the ovarian follicular development and production of mature oocyte. In the present study, the cyclic changes of LH between proestrus and diestrus were absent in HA feeding mice (Figure 3A), and the ovarian follicle reserve was lower in HA feeding mice compared with NA mice (Figure 1I). However, fertility test revealed that the pups produced in mice fed the high‐fat diet were not impaired in the first pregnancy (Figure 2A). On the other hand, TRF improves the reproductive outcome during the fertility test through the three pregnancies (Figure 2D). These results suggested that parameters related to reproductive process were differentially affected by high‐fat feeding and TRF. The reproductive process of mammal animals is quite complex and is of high plasticity and flexibility through delicate mechanisms,^45^, ^46^ and those mechanisms, at least, require the cooperation among the hypothalamus, pituitary and the gonadal tissues, which is usually called the “reproductive axis.”^47^, ^48^ Therefore, the reproductive function is controlled by a variety of regulators in the tissues of hypothalamus, pituitary, and ovary. Metabolisms was known to be the important factors influencing the function of hypothalamus‐pituitary‐gonadal axis.^49^, ^50^ Meanwhile, some of the metabolic factors (e.g. adiponectin^35^, ^51^, ^52^ and IGF1^50^, ^53^, ^54^) would exert their effects directly on ovarian development to influence reproduction, and others (e.g. leptin^50^, ^55^, ^56^) might exert their effects on reproduction both via hypothalamus and ovaries. Both HFD feeding and TRF dietary regimen are important dietary pattern that will affect health and metabolism, and it is possible that HFD feeding and TRF dietary regimen exert their effects on reproduction via different pathways. Given that the reproductive process is controlled by muti‐level regulators, and is regulated differentially by HFD feeding or TRF dietary regimen, it is reasonable that some of the reproductive indices were affected by HFD feeding, and some of the parameters were affected by TRF regimen.

In the present study, our results revealed that TRF improved metabolic health in mice without negative effects on their estrus cycles (Figure 1F). In line with this, TRF increased circulating 17β‐estradiol levels during proestrus and the number of antral follicles in HT mice compared to HA mice. In human and mice, ovarian *Foxo3a* expression levels are associated with puberty onset and fertility. Foxo3a‐/‐ mice exhibited a marked decline of fertility by loss of functional follicles.^57^ FOXO3A also plays an intra‐oocyte role controlling follicular activation and development.^58^ In the present study, we found that TRF increases ovarian *Foxo3a* expression levels via the activation of FGF21 signaling (Figure 1L,K), which suggested TRF improved the ovarian disfunction through the regulation of FOXO3 levels. Various studies have focused on extending the reproductive span by chronic caloric restriction.^7^, ^59^, ^60^ however, the reproductive capacity was shown to be impaired by a prolonged caloric restriction, resulting in subfertility or even infertility.^7^, ^60^ In the present study, TRF increased the number of offspring and successful pregnancies rate in mice fed both the chow diet and high‐fat diet (Figures 2D,G), which was coincident with the greater number of ovarian reservations found in the TRF groups (Figure 1I). Since the ovarian reservation is closely related to lifetime fertility in female, the greater ovarian reserve in the TRF mice suggested that TRF would exert a long‐term effect on fertility in female mice.

Prior work has demonstrated that liver metabolism integrated nutritional status and reproductive cycle,^54^, ^61^ thereby suggesting a possible hepatokine linking metabolism and reproduction. Time‐restricted feeding pattern involves an intermitting fasting period which changed the circulating levels of FGF21.^62^ The hepatokine FGF21, known as a key metabolic regulator and endocrine signaling through central and peripheral pathways,^22^, ^23^, ^26^, ^29^ can promote the metabolic flexibility. In mice, plasma FGF21 has been found to be increased after 6‐h fasting.^63^ Interestingly, FGF21 exhibits a day‐night circadian oscillation both in rodents and human. In healthy female, plasma FGF21 levels have a day‐night variation pattern and are affected by daily food intake.^62^, ^64^ In mice, the physiologic level of FGF21 can act on hypothalamus to regulate metabolism and circadian behavior.^22^ In female mouse, FGF21 levels underwent the tetradian oscillatory during estrus cycle,^29^ suggesting a possible role of FGF21 in estrus cyclicity. Time‐restricted feeding, an eating pattern without caloric restriction, increased daily fasting time. In this study, TRF increased the circulating levels of FGF21 in fed state both at proestrus and diestrus. Under the TRF, circulation FGF21 levels still exhibits circadian oscillation but with a greater vibration amplitude compared with *ad libitum*,^62^ suggesting that TRF mice has a stronger metabolic activity.

FGF21 has recently been shown to be able to promote neurite outgrowth in vitro^65^ and the FGF21 receptor KLB, plays a key role in GnRH neuron homeostasis.^31^ Consistently, we found that recombinant FGF21 increased GnRH secretion in the immortalized GnRH cell line (GT1‐7) in a dose‐dependent manner (Figure 3I) and depend on ERK_1/2_ pathway (Figure 3J). *In vivo*, we found that TRF increased circulating FGF21 levels and hypothalamic *Gnrh* mRNA levels at proestrus (Figure 3D), and the greater *Gnrh* expression might in turn induce the LH surge (Figure 3C). Given that liver accounts for more than 80% of the FGF21 in circulation,^29^, ^35^, ^66^ liver‐specific knockout of FGF21 mouse model was established in order to test the role of FGF21 in the regulation of fertility by TRF. Interestingly, results revealed that loss of liver FGF21 abrogated the beneficial effects of TRF on estrus cyclicity, follicular development, serum E2 level, and gene expressions relating to ovarian follicular growth and steroidogenesis, suggesting the mediatory role of liver FGF21 in the control of TRF benefits. In fact, this is not the first evidence elucidating the role of FGF21 in the control of fertility by dietary regimen. Our previous research found that liver FGF21, serving as a metabolic signal of diet composition, mediated the effects of macronutrients balance on the follicular reservation and reproductive span. Conversely, prior work observed that female mice with FGF21 overexpression were infertile via central suppression of kisspeptin signaling and GnRH release.^23^ However, mice engineered to transgenically overexpress FGF21 had superhysiologic levels of FGF21, and the average level of circulating FGF21 in this study was ∼100 times lower than that in mice transgenically overexpressing FGF21. In both rodents and humans, fasting can delay puberty and is accompanied by increased levels of serum FGF21.^67^ Fasting in a similar manner to caloric insufficiency which causes a rapid body weight loss and a strong stress response, and is not a normal physiologic condition. Meanwhile, disruption of the FGF21 signaling by deleting β‐klotho resulted in an abnormal estrus cycle including a blunted LH surge at the estrus stage and a reduced pregnancy rate.^31^ Overall, FGF21 may act as a critical player in modulating female fertility subjected to various nutritional situations.

In human, FGF21 levels are increased in response to time‐restricted feeding, thereby showing day‐night variation pattern in young healthy females, although this increase diminished at the end of a 24‐h fast.^64^ However, plasma FGF21 levels were not altered after a 2‐day fast in obese male.^68^ Therefore, the application of targeting circulating FGF21 by TRF to improve metabolic homeostasis and reproductive fitness still awaits further validation in human clinical trials.

## 5 STUDY LIMITATIONS

There are several strengths to this study, but there are also limitations. First, adipose tissue is the main target of FGF21 under physiological conditions, and further investigations are needed to assess the impact of adipose tissue metabolism in the control of reproduction by FGF21. Second, our data provide significant evidence that time‐restricted feeding improved the female reproduction, and liver FGF21 played an important role during this process, however, this TRF effect on human reproduction is still unclear, and the application of targeting circulating FGF21 by TRF to improve metabolic homeostasis and reproductive fitness still awaits further validation in human clinical trials.

## 6 CONCLUSIONS

In this study, our findings indicate that time‐restricted feeding improves the reproductive function of female mice and liver FGF21 signaling plays a key role in this process (Figure 5). Taken together, our findings provide evidence that during TRF, FGF21 may act as a critical player in modulating female fertility and provide novel insights into the dietary intervention on obesity or infertility interventions.

## CONFLICT OF INTEREST

The authors declare no conflict of interest.

## AUTHOR CONTRIBUTIONS

D. Wu, Y. Zhuo, and L. Hua conceived and designed the research; D. Wu, L. Hua, Y. Zhuo, and B. Feng wrote the paper; L. Hua, Y. Zhuo, L.S. Huang, J. Li, and X. Han performed the research and analyzed the data; X.M. Jiang, L.Q. Che, T. Luo, Z.F. Fang, S.Y. Xu, and Y. Lin contributed to the analysis and manuscript preparation; All authors read and approved the final manuscript.

## FUNDING

This study was supported in part by National Key R&D Program of China (Grant 2018YFD0501005), National Natural Science Foundation of China, PR China (Grant 31772616) and the 111 Project (D17015). The funding source had no role in study design, collection, analysis, and interpretation of data, writing of the report, or in the decision to submit the paper for publication.

## AVAILABILITY OF DATA AND MATERIALS

The data that support the findings of this study are available from De Wu upon reasonable request.

## ETHICS APPROVAL AND CONSENT TO PARTICIPATE

All animals received humane care according to the criteria outlined in the Guide for the Care and Use of Laboratory Animals. All animal experiments were conducted in accordance with the guidelines of the Animal Care and Ethics Committee of Sichuan Agricultural University.

## Supporting information

12‐week‐old female WT (FGF21^fl/fl^) and FGF21 LKO mice were either *ad libitum* or fasted 24 h (n = 6/group), and liver (A), eWAT (B), iWAT (C), BAT (D) and muscle (E) *Fgf21* mRNA levels were analyzed by RT‐PCR. Statistical significance was evaluated by the 2‐way ANOVA test or with the Tukey's test for multiple comparisons to determine differences among each group, where appropriate. *** *P* < 0.001.
