# Supplementary material for: Time‐restricted feeding improves the reproductive function of female mice via liver fibroblast growth factor 21
Source: Clin Transl Med. 2020 Oct 4;10(6):e195. doi: 10.1002/ctm2.195 (PMC7533054; doi:10.1002/ctm2.195)
Supplement: Supplementary file 1 — 12‐week‐old female WT (FGF21fl/fl) and FGF21 LKO mice were either ad libitum or fasted 24 h (n = 6/group), and liver (A), eWAT (B), iWAT (C), BAT (D) and muscle (E) Fgf21 mRNA levels were analyzed by RT‐PCR. Statistical significance was evaluated by the 2‐way ANOVA test or with the Tukey's test for multiple comparisons to determine differences among each group, where appropriate. *** P < 0.001. [file CTM2-10-e195-s001.docx]

**Supplementary Table 1.** Sequence of primers used for qPCR.

| **Genes** | **Forward** | **Reverse** |
| --- | --- | --- |
| ***Actin*** | F:GGCTGTATTCCCCTCCATCG | R:CCAGTTGGTAACAATGCCATGT |
| ***Amh*** | F:ATCTGGCTGAAGTGATATGG | R:CAGGGTATAGCACTAACAGG |
| ***Bmp15*** | F:GCACGATTGGAGCGAAAATG | R:CGTACGCTACCTGGTTTGATGC |
| ***Cdh1*** | F:TTGGTGTGGGTCAGGAAATC | R:GTGTCCCTCCAAATCCGATAC |
| ***Cyp11a1*** | F:GGCCCAATTTACAGGGAGAAG | R:CACCAGGGTACTGGCTGAAG |
| ***Cyp19a1*** | F:CGGGCTACGTGGATGTGTT | R:GAGCTTGCCAGGCGTTAAAG |
| ***Foxo3a*** | F:ACTGAGGAAAGGGGAAATGG | R:CAAAGGTGTCAAGCTGTAAACG |
| ***Fshr*** | F:TCTGGGCCACTCGTTTTACAC | R:TTGCATTCCAGTTGCATGGC |
| ***Inha*** | F:TGAACCAGAGGAGGAAGATGTCTC | R:TCTGGCAGCTGGCTGGTC |
| ***Lhcgr*** | F:ACGAGACGCTTTATTCTGCCA | R:AGGGGTACTTTGAAGGCAGC |
| ***Nobox*** | F:CTATCCTGACAGTGACAAACGCC | R:CACCCTCTCAGCACCCTCATTAT |
| ***Rspo2*** | F:GCGGGTGTCGGCAAACTTTTTC | R:ATCTGGGGCTCGGTGTCCATAATAC |
| ***Star*** | F:TCCCTCGCAGGACCTTGATCT | R:TGGATGGGTCAAGTTCGACG |
| ***Wnt2*** | F:CACCAGTTCCGCCAGCAT | R:GAGGACCCGGCCAAAGA |
| ***Gnrh*** | F:AGCACTGGTCCTATGGGTTG | R:GGGGTTCTGCCATTTGATCCA |
| ***Kiss-1*** | F:CTCTGTGTCGCCACCTATGG | R:TTCCCAGGCATTAACGAGTTC |

**Abbreviations:** Amh, anti-mullerian hormone; Bmp15, bone morphogenetic protein 15; Cdh1, cadherin 1; Cyp11a1, cytochrome P450 family 11 subfamily A member 1; Cyp19a1, cytochrome P450 family 19 subfamily A member 1 (aromatase); Foxo3, forkhead box O3; Fshr, follicle stimulating hormone receptor; Inha, inhibin a; Lhcgr, luteinizing hormone/chorionic gonadotropin receptor; Nobox, NOBOX oogenesis homeobox; Rspo2, R-spondin 2; Star, steroidogenic acute regulatory protein; Wnt2, Wnt family member 2.

**Supplementary Table 2. The effect of time-restricted feeding on pregnancy ratio**

| Items % | NA (n=20) | NT (n=20) | HA (n=21) | HT (n=20) |
| --- | --- | --- | --- | --- |
| Pregnancy 1 | 85.00 | 100.00 | 95.23 | 95.00 |
| Pregnancy 2  Pregnancy 3 | 80.00  50.00 | 100.00  65.00 | 85.71  57.14 | 85.00  75.00 |

NA means mice eating a normal chow diet with *ad libitum*, NT means mice eating a normal chow diet with time-restricted access to food, HA means mice eating a high-fat diet with *ad libitum*, and HT means mice eat a high-fat diet with time-restricted access to food.
